# The Role of Essential Oils against Pathogenic *Escherichia coli* in Food Products

**DOI:** 10.3390/microorganisms8060924

**Published:** 2020-06-18

**Authors:** Paulo E.S. Munekata, Mirian Pateiro, David Rodríguez-Lázaro, Rubén Domínguez, Jian Zhong, Jose M. Lorenzo

**Affiliations:** 1 Centro Tecnolóxico da Carne de Galicia, rúa Galicia n◦ 4, Parque Tecnolóxico de Galicia, San Cibrao das Viñas, 32900 Ourense, Spain; paulosichetti@ceteca.net (P.E.S.M.); mirianpateiro@ceteca.net (M.P.); rubendominguez@ceteca.net (R.D.); 2 Microbiology Division, Department of Biotechnology and Food Science, Faculty of Sciences, University of Burgos, 09001 Burgos, Spain; drlazaro@ubu.es; 3 Integrated Scientific Research Base on Comprehensive Utilization Technology for By-Products of Aquatic Product Processing, Ministry of Agriculture and Rural Affairs of the People’s Republic of China, Beijing 100125, China; 4 National R&D Branch Center for Freshwater Aquatic Products Processing Technology (Shanghai), Shanghai Engineering Research Center of Aquatic-Product Processing and Preservation, College of Food Science & Technology, Shanghai Ocean University, Shanghai 201306, China; jzhong@shou.edu.cn; 5 Área de Tecnología de los Alimentos, Facultad de Ciencias de Ourense, Universidad de Vigo, 32004 Ourense, Spain

**Keywords:** *E. coli* O157:H7, Shiga toxin, terpenes, virulence regulation, antimicrobial mechanism, meat, dairy products, vegetables, food safety

## Abstract

Outbreaks related to foodborne diseases are a major concern among health authorities, food industries, and the general public. *Escherichia coli* (*E. coli*) is a pathogen associated with causing multiple outbreaks in the last decades linked to several ready to eat products such as meat, fish, dairy products, and vegetables. The ingestion of contaminated food with pathogenic *E. coli* can cause watery diarrhea, vomiting, and persistent diarrhea as well as more severe effects such as hemorrhagic colitis, end-stage renal disease, and, in some circumstances, hemolytic uremic syndrome. Essential oils (EOs) are natural compounds with broad-spectrum activity against spoilage and pathogenic microorganisms and are also generally recognized as safe (GRAS). Particularly for *E. coli*, several recent studies have been conducted to study and characterize the effect to inhibit the synthesis of toxins and the proliferation in food systems. Moreover, the strategy used to apply the EO in food plays a crucial role to prevent the development of *E. coli*. This review encompasses recent studies regarding the protection against pathogenic *E. coli* by the use of EO with a major focus on inhibition of toxins and proliferation in food systems.

## 1. Introduction

Food safety is a major issue with severe and life-long implications to public health when food contaminated with pathogenic microorganisms is consumed [1]. Among the main foodborne pathogens, *Escherichia coli* have been standing out due to recurrent outbreaks every year. Some of the food products related to this pathogenic bacterium are clove sprouts, ground bison meat, chicken salad, beef patties, raw milk cheese, and seafood sauce [2,3].

The intestinal pathogenic *E. coli* groups are enteropathogenic (EPEC); Shiga-toxin-producing (STEC, also known as enterohemorrhagic—EHEC), enteroinvasive (EIEC, closely related to *Shigella* spp.), enteroaggregative (EAEC), enterotoxigenic (ETEC), and diffusely adherent (DAEC). The common symptoms associated with these groups of *E. coli* are diarrhea usually associated with contamination levels in the range 6–10 log CFU. The exceptions are STEC/EHEC and EIEC groups, which have the lowest infectious dose among pathogenic *E. coli* (down to 1000 CFU) [4,5,6]. The production of toxins is a common characteristic that can cause other symptoms in addition to watery diarrhea: persistent diarrhea (EAEC), abdominal pain and vomiting (EPEC), hemolytic uremic syndrome (HUS), end-stage renal disease (ESRD), and hemorrhagic colitis (STEC) [5,7]. Major attention has been given for the STEC group due to the severity of symptoms and after-effects that can occur by the ingestion of food of animal (meat, fish, and milk, for instance) and vegetable (such as broccoli, lettuce and kale) origin as well as water. The symptoms include Shiga intoxication, which includes intestinal discomfort to more complicated clinical manifestations that can evolve to death [7,8]. It is also relevant mentioning that pathogenic *E. coli* also causes extraintestinal infections. Some of the extraintestinal *E. coli* related to human infections are the uropathogenic (UPEC), meningitis/sepsis-associated (MNEC), necrotoxic (NTEC), which urinary tract infections, meningitis, and necrosis, respectively [6]. The extraintestinal pathogenic *E. coli* will not be considered in this review.

EHEC is a resident bacterium of cattle gastrointestinal (GI) tract. In this sense, fecal shedding is a meaningful source of contamination of food and water. Once EHEC reaches the human GI tract, two main virulence strategies can take place: (1) the attaching and effacing lesions (AEL) on enterocytes and (2) production of Shiga toxins (Stx-1 and Stx-2). AEL process initiates from the migration of EHEC from intestinal lumen towards the surface of enterocyte. Then, EHEC activates the expression of the enterocyte effacement island that encodes the *ler* (master regulator), a type 3 secretion apparatus, translocator proteins EspA and EspB, and an adhesin, intimin, and its own translocated intimin receptor. The EHEC can form and preserve the AEL (a pedestal-like structure) between the bacterial cell and the enterocyte. In the case of Stx, its absorption by enterocytes can lead to several symptoms. Stx is released from bacterial cell lysis and causes hemorrhagic colitis. Moreover, Stx can reach the bloodstream and cause severe effects. The most common are HUS, thrombocytopenia (low platelet count), hemolytic anemia (lysis of red blood cells), and acute renal failure [4,9].

Regarding the structural characteristics and mechanisms of action in the human body, the Stx is composed of an A sub-unit non-covalently bound to five B sub-units. The A sub-unit acts on the inhibition of protein synthesis compromising the functionality of ribosomes. The B sub-unit is responsible for the attachment of Stx to globotriaosylceramide (Gb3) receptor located on the external side of eukaryotic cells associated with the intestinal mucosa and kidney. Stx can be classified in two groups according to immunological responses: Stx-1 (very similar to the Shigella Stx structure, amino acid sequence, and toxicity) and Stx-2 (almost half of similarity to Stx-1). Another relevant aspect is the difference in toxicity between Stx-1 and Stx-2. The prevalence of *stx1* is greater than that of *stx2* and some strains harbored both *stx1* and *stx2* genes [4,10].

**Figure 1:**
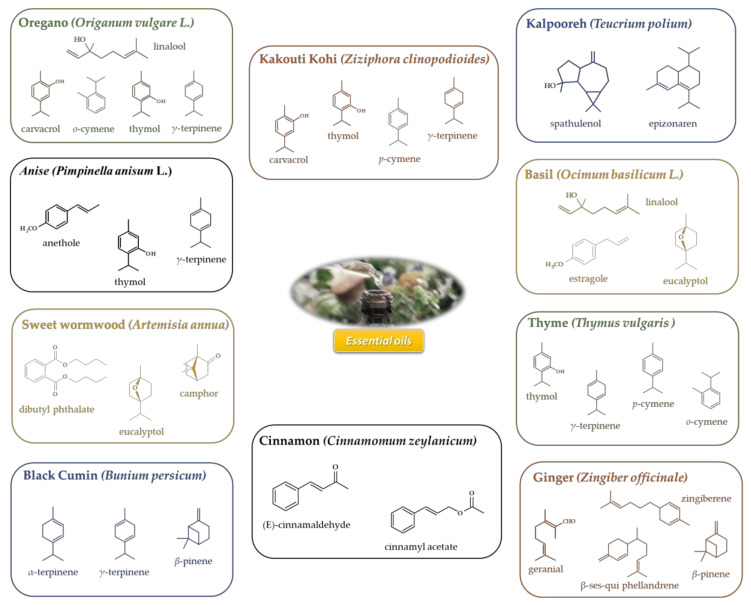
Selected essential oils (Eos) and their main components.

In order to improve the protection against the growth and production of toxins by pathogenic *E. coli* and other microorganisms, the food industry uses antimicrobial compounds (food preservatives). These compounds are restricted to specific limits due to potential health risks [11,12]. In this line of thought, the search for suitable alternatives is a major topic of research and development in the food area, particularly for compounds from natural sources [13,14,15,16,17]. Essential oils (EOs) are natural compounds found in several plants that are involved in secondary metabolism. Essentially, EOs are mixtures of volatile compounds with odorous properties (insect repellent or seasoning food, for instance). EOs are comprised of a complex mixture of terpenes, sesquiterpenes polyphenols, lactones, esters, alcohol, and others [13,18,19,20,21]. Selected sources and their main compounds are displayed in Figure 1.

The effectiveness of EOs as antimicrobial agents in food products has been reported in several studies against several spoilage and pathogenic microorganisms: meat and meat products [22], dairy products [23], fish and fish products [24], and vegetables [25]. Moreover, the EOs have anticancer potential (supported by in vivo studies) [26] and are considered as generally recognized as safe (GRAS) [27]. The safety of EOs and their main components (expressed as median lethal dose—LD_50_) is derived from safety assessment tests (in vivo) that indicated no toxic effect in mammalians, in most cases. Thyme, oregano, clove, cinnamon, thymol, carvacrol, linalool, eugenol, and *p*-cymene are examples of Eos, whose main components considered as GRAS. The high LD_50_ value of an EO or isolated compound supports their application as food preservatives [28]. Additionally, interesting advances about the effect and use of EOs against the pathogenic *E. coli* (in vitro and in food) have been reported but the compilation of these recent advances has not been done yet. In this sense, the present review will discuss the role of essential oils against pathogenic *E. coli* (especially O157:H7 strain) and the most successful strategies to apply these compounds in the food system.

## 2. Mechanisms Related to Protective Effect of EOs Against Pathogenic *E. coli*

**Figure 2:**
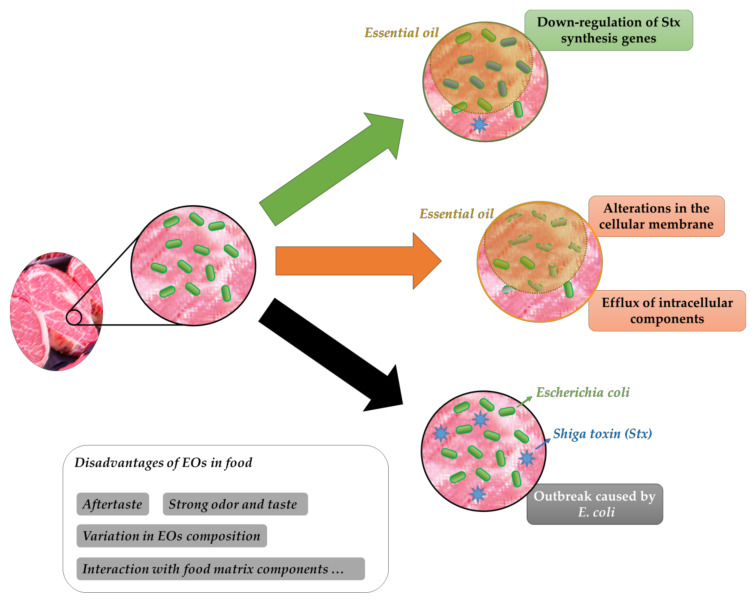
Influence of EOs on pathogenic *E. coli* and some disadvantages of EOs on food.

In order to clarify the protection related to EOs in food systems against *E. coli*, it is relevant to understand the influence of EOs in the metabolism, stability of vital structural components, and genetic expression related to pathogenicity (Figure 2).

### 2.1. Regulation of Virulence-Related Genes

The genes encoding the Stx are located in several lambdoid bacteriophages, which are induced by phage gene promoters and the release of Stx [10]. The first approach to understand the influence of EOs in *E. coli* is the study of the influence of concentrations up to the minimal inhibitory concentration (MIC). Increasing the EO concentration up to its MIC value causes a gradual inhibition of metabolism. In this condition, it is possible to clarify and understand the antipathogenic mechanisms of EOs. It is relevant mentioning that recent studies characterized the MIC value of EOs against pathogenic *E. coli* to be in the range in the range of 0.025 and 0.1% [29,30,31,32]. The inhibition of toxin synthesis when pathogenic *E. coli* are exposed to EOs is presented in Table 1.

The study carried out by Mith et al. [33] evaluated the influence of oregano EO and carvacrol (one of the main components in oregano EO) on the synthesis of Stx. The authors observed that both oregano EO and carvacrol induced a down-regulation in the expression of *ler*, *stx2B*, and *luxS* (associated with cell-to-cell communication and pathogenicity) genes. The authors also observed that a concentration-dependent effect was observed after increasing the concentration of both oregano EO and carvacrol from 0.05 to 0.08 µL/mL, especially for *luxS* expression. Another relevant outcome was reported by Sheng et al. [29]. In this case, the authors observed that cinnamon EO down-regulated *hfq*, *luxS*, *qseB*, *qseC*, and *stx2* when adding 0.75 MIC in the culture medium. Another interesting outcome obtained from this experiment was the down-regulation of *stx2* expression during both lag and exponential phase with 0.75 and 1 MIC of cinnamon oil. However, the same result was not observed for *E. coli* exposed to 0.25 and 0.50 MIC wherein the influence of concentration was not clear. While 0.25 MIC was associated with a slight inhibition, bacteria treated with 0.5 MIC displayed the same levels of *stx* expression observed on control (non-treated) *E. coli*.

The encapsulation of EO have shown promising effects regarding the protection against the expression of virulence-related genes [3]. This approach was evaluated with *Zataria multiflora* EO on the expression of *stx2A*. The down-regulation was concentration-dependent between 0.25 and 1 MIC for both free and nanoliposomes loaded with *Zataria multiflora* EO. Interestingly, this effect was more intense on nanoliposomes than in free form.

The down-regulation of AEL-related genes (*ler*, *espD*, *escJ*, *escR*, and *tir*) was observed after *E. coli* was exposed to clove EO and eugenol (a major component of clove EO) [31]. However, the expression of genes related to cell-to-cell communication and Stx synthesis was not influenced by both clove EO and eugenol. In a similar way, the experiment carried out by Mahmoudzadeh [32] did not obtain down-regulation of *stx1A* and *stx2A* in the culture medium, which was in fact up-regulated by 0.03%.

To the best of our knowledge, only a single study explored the influence of EOs in the expression of genes related to virulence in food systems [32]. In this experiment, ground beef was inoculated with *E. coli* O157:H7 (10^9^ CFU/g meat) and the inhibitory effect of *Carum copticum* EO in the expression of *stx1A* and *stx2A* genes was observed on days 2 (0.5 MIC of EO) and 5 (0.75 MIC of EO) of storage. After this, the inhibitory effect was reduced for both treatments with *C. copticum*.

### 2.2. Antibacterial Mechanisms

Using EOs at MIC levels causes a significant effect on *E. coli* cells. In order to characterize the bactericidal effect of EOs, many studies reported that morphological alterations in the membrane are the main and most meaningful bactericidal effect on *E. coli*. Once this effect takes place, the functionality of the membrane is drastically altered, which alters vital metabolic processes [34,35,36,37]. The characteristic bacilli shape with uniform surface is converted to a diffuse and non-uniform shape. In other words, integrity is gradually reduced, and cells begin to swell. In this condition, an efflux of intracellular components takes place and further exposing the cells to the effects the EOs cause the collapse of surface and lysing of cells [37].

The study carried out by Oussalah et al. [36] characterized the mechanisms involved in the inactivation of pathogenic *E. coli* (O157:H7) by Spanish oregano (*Corydothymus capitatus*), Chinese cinnamon (*Cinnamomum cassia*), and savory (*Satureja montana*) EOs. The authors observed that the efflux of intracellular components and the loss of intracellular homeostasis was observed after exposure of *E. coli* cells to all three EOs. This condition was characterized by the reduction of intracellular pH, an imbalance in the intra-and extracellular adenosine triphosphate (ATP) levels. Another relevant outcome was the reduction of ATP levels without the release to extracellular medium when *E. coli* was exposed to Chinese cinnamon. According to authors, this EO induced the hydrolysis of ATP in a condition of reduced membrane damage (pore formation rather than loss of membrane integrity).

A similar experiment was carried out by Patra et al. [34], who evaluated the effect of *Enteromorpha linza* L. EO on pathogenic *E. coli* (ATCC 43889 and 43890). This EO disrupted the membrane and caused the efflux of intracellular components. Interestingly, the authors observed that the tolerance to NaCl was also reduced by the exposure to *Enteromorpha linza* L. EO. This result is an important advance to understand the influence on products where salt plays a major role in the processing and storage stability. Another relevant effect against *E. coli* (O26, O45, O103, O104, O111, O121, O145, and O157) was associated with carvacrol (one of the major components of oregano EO) [35]. In addition to the disruption of cell membrane and reduction of intracellular ATP level and altered membrane permeability and integrity, the authors observed that carvacrol reduced the adherence to human intestinal cells.

An interesting outcome was reported by Moghimi et al. [38] who studied the influence of nanoemulsification of *Thymus daenensis* EO on pathogenic *E. coli*. The authors indicated that the nanoemulsified EO at MIC level (0.4 mg/mL) greatly improved the leakage of intracellular components (nucleic acid, proteins, and potassium) in comparison to the free EO at MIC level (4.0 mg/mL). This difference was related to the intense deformation and disruption of the membrane of *E. coli* treated with nanoemulsified EO. Conversely, a slight shrinkage was observed when *E. coli* was exposed to the free EO. It is also relevant mentioning that the bactericidal effect of EOs on pathogenic *E. coli* can also be influenced by microorganism physiological state [39].

## 3. Application of EOs in Food Systems

### 3.1. Meat and Meat Products

Among the many possible strategies to use natural EOs to reduce the counts of *E. coli* in meat and meat products, the sole and combined approaches have been showing promising results (Table 2). First, the direct addition of EOs as an ingredient is a common strategy explored by many authors. Second, the addition of EOs to marinate solutions is another relevant strategy. Third, the incorporation of EOs in the structure of films and coating layers are an effective system to kill *E. coli* in meat and meat products.

Regarding the first strategy, the study carried out by Ed-Dra et al. [40] explored the addition of *Mentha suaveolens* EO (2, 5, and 10 mg/g) as a natural preservative in fresh turkey sausages. According to authors, a reduction of 1.2 log CFU/g in *E. coli* after 48 h of storage at 4 °C was obtained using 10 mg EO/g. The authors also indicated that at this concentration significant reductions in the counts of *E. coli* (ATCC 25922) were observed after 6 h of processing in this concentration. Moreover, a concentration-dependent effect was observed after 24 and 48 h. Another related outcome related was reported for the use of clove bud oil in cooked beef patties [41]. This EO reduced the counts of *E. coli* O157:H7 by 0.3 log CFU/g in patties in comparison to control samples. In the same line, the use of *Ziziphora clinopodioides* EO in beef patties gave a reduction of 5 log CFU/g after seven days of storage at 4 °C [42]. The study carried out by Bortolotto et al. [43] explored the use of garlic EO in combination with allyl isothiocyanate as preservative in fresh pork sausage. The authors observed that the *E. coli* O157:H7 count was reduced by using 125 and 250 ppm of garlic EO and 250 ppm allyl isothiocyanate after 20 days of storage (reduction of 0.7 and 1.6 log CFU/g after 20 days in relation to control samples). However, reducing allyl isothiocyanate to 125 ppm did not induce significant reductions in *E. coli* counts.

In the same line, the addition of isolated compounds from natural EO was also associated with an additional protective effect in salami [44]. In this case, the authors evaluated the impact of allyl isothiocyanate (3.125 and 6.25 µL/100 g) in combination with the phenolic compound *o*-coumaric acid (375 and 750 mg/100 g) during the processing of salami (35 days). The expected competitive effect of starter culture on the counts of *E. coli* O157:H7 was observed by means of gradual reduction over time. Moreover, the addition of this combination of natural compounds led to a more intense reduction of *E. coli* counts. According to authors, the salami produced with the combination of 62.50 µL/L of allyl isothiocyanate with 7000 mg/kg of *o*-coumaric acid reduced the *E. coli* counts below the detection limit of the test (0.9 log CFU/g) from day 21 until the end of processing. A similar outcome, but less intense, was observed using 31.25 µL/L of allyl isothiocyanate with 3500 mg/kg of *o*-coumaric acid. It is worth mentioning that the production of emulsions with EO was also associated with an inhibitory effect on *E. coli* in chicken pâté [45]. This effect (reduction of 1.5 log CFU/g) was obtained using 12 g oregano EO emulsion/kg after three days of storage.

The use of EOs in marinating solutions to improve the safety of fresh chicken meat against *E. coli* requires major attention [46,47]. This consideration is supported by the outcome of the recent study carried out by Karam et al. [46]. The authors indicated that the *E. coli* count was reduced by 0.8 log CFU/g after 21 days of storage after marinating with thymol and carvacrol mixture. In the same line, Van Haute et al. [47] did not observe any impact in the counts of *E. coli* on chicken skin marinated with cinnamon, oregano, and thyme essential oils.

The incorporation of EOs in the coatings and films is an interesting approach that can improve the safety against *E. coli*. The inclusion of anise EO (1, 1.5, and 2%) in an edible chitosan film induced the reduction on the counts below the detection limits of the method of this bacterium in a chicken burger after three days of storage [48]. A similar effect was observed when authors used the chitosan film with 0.5% of anise EO. In this treatment, the reduction of *E. coli* O157: H7 count below detection limits was obtained after six days of storage.

In the same line, the film developed by Zhang et al. [49] with fish skin gelatin, ZnO and ginger EO (80%, EO:protein in film) inhibited the growth of *E. coli* during nine days at 4 °C (reductions from 2 to 6 log CFU/g). The authors observed a concentration-dependent effect wherein increasing the concentration of ginger EO in film (from 10 to 80%, EO:protein ratio in film) enhanced effect against *E. coli* growth in comparison to both controls (not packaged and packaged without antimicrobial compounds). Likewise, coating meat with an active solution can also improve the protection against *E. coli*. The coating produced by Hernández-Hernández et al. [50] with microencapsulated oregano Mexican oregano and basil EO mixture inhibited the growth of *E. coli* 0157:H7 in fresh pork meat during 28 days at 4 °C. The authors obtained 5.7 log CFU/cm^2^ reduction in comparison to uncoated samples.

Each strategic use of EOs as preservatives in meat and meat products against *E. coli* is largely influenced by the strategy to incorporate or apply them. In the case of films and coating layers, the protection is greatly improved by causing an important reduction in *E. coli* counts throughout the storage period. Conversely, marinating seems to be less effective to improve the protection against *E. coli* in comparison to other approaches (additive, film, and coating layer).

### 3.2. Fish and Fish Products

The protective effect of EO against *E. coli* in recent studies is presented in Table 3. The approaches used in these studies are centered on direct use, dispersing in a solution (dipping or spraying) and in the composition of coating layers. An interesting approach consisting of spraying the EO (oregano and thyme) on the surface of fish [51]. This approach resulted in a reduction of *E. coli* O157:H7 count up to 1.2 log CFU/g in rainbow trout fillets after four days at 4 °C. It is worth noting that the authors obtained this outcome by combining the EOs with vacuum and modified atmosphere packaging (MAP). However, the packaging under aerobic conditions did not indicate significant differences between non-treated (control) and EO-treated samples.

The study carried out by Abdeldaiem et al. [52] explored the use of rosemary, cinnamon, fennel, and cardamom EOs to prevent the proliferation of *E. coli* in carp flesh fingers. The authors observed that the inhibitory effect was obtained for all tested EOs, particularly in the first six days of storage at 4 °C. In the same line, Al-Hajj et al. [53] evaluated the impact of *Pulicaria inuloides* EO on *E. coli* inoculated in Kachlan (*Trachinotus ovatus*, Linnaeus) fillets and obtained reduction of 1 log CFU/g.

Another relevant approach to improve the safety against *E. coli* in fish is using a coating layer. This approach was explored in rainbow trout fillets using *Zataria multiflora* and *Bunium persicum* EOs [54]. Both EOs were associated with reductions in *E. coli* O157:H7 counts up to 1 log CFU/g, especially using the 1.0% of *Zataria multiflora* and *Bunium persicum* EOs. A similar outcome was obtained in a further experiment using *Zataria multiflora* Boiss EO in a coating solution in rainbow trout fillets stored at 4 °C for 16 days [55]. Finally, an experiment with clove EO in a coating layer indicated reductions between 3 and 5 log CFU/g for *E. coli* (PTCC 3315) in silver carp fillets after 16 days of storage at 4 °C [56].

The protective effect of EO against *E. coli* is limited to reductions in the range of 1–2 log CFU/g in seafood. The use of coating layers seems the most promising strategy, but further experiments are necessary to optimize the use of this approach.

### 3.3. Milk and Dairy Products

The use of EOs against pathogenic *E. coli* in milk and dairy products is presented in Table 4. The main strategies to add EOs in milk and dairy products are to use them as a food additive or incorporate them in coatings/films. In relation to the EOs added as food additives, a recent study explored the effect of free and emulsified thymol concentration on *E. coli* O157:H7 in milk [57]. The authors indicated that free and emulsified thymol were both effective to reduce the *E. coli* counts after 48 h at 21 °C on skim, 2% fat, and whole milk. The markable reductions associated with thymol were observed using 4.5 g/mL of thymol EO in both free and emulsified forms (around 6 log CFU/mL). However, the level of fat was indicated as a factor of major importance. According to authors, the decrease in *E. coli* counts observed in the beginning of the incubation period (from 0 to 24 h) was more intense in 2% fat milk than in whole milk using 4.5 g/L.

The inhibitory effects of EOs in pathogenic *E. coli* was also observed in dairy products. For instance, the inclusion of *Teucrium polium* EO (75 and 150 mg/L) in the formulation of Kishk (a fermented milk produced and consumed in the Indian subcontinent and Middle East) was associated with a gradual decrease in *E. coli* O157:H7 count [58]. During refrigerated storage, the authors obtained reductions of 0.8 and 1.3 log CFU/g for 75 and 150 mg/L, respectively. A similar outcome was observed for Doogh (an Iranian yogurt drink) produced with *Ziziphora clinopodioides* EO [59]. According to the authors, a slight reduction (up to 3 log CFU/mL) in *E. coli* counts in comparison to control samples after three days of refrigerated storage was shown.

The use of EOs as food additives was associated with a slight effect in the study carried out by Ehsani et al. [60] with Iranian white cheese. In this case, the authors added *Bunium persicum* EO (1 and 2%) to cheese and obtained a reduction of 0.6 log CFU/g of *E. coli* O157:H7 counts in comparison to untreated cheese. Another related study indicated that thyme and clove EOs reduced *E. coli* O157:H7 in Kariesh cheese during storage [61]. After 14 days of storage different outcomes were obtained for each EO: clove EO (1%) caused a reduction of 1.8 log CFU/g, whereas the reduction on cheese produced with thyme EO (1%) was 6 log CFU/g.

Differently, the effect of EOs against pathogenic *E. coli* can be improved by incorporating them in coatings and films. This outcome was reported by Kavas et al. [62] who observed a marked decrease in *E. coli* O157:H7 counts in Kashar cheese (a semi-hard Turkish cheese) produced with thyme and clove EO (1.5%) during 60 days of refrigerated storage. Moreover, the authors also argued that the thyme EO displayed a higher antimicrobial effect than clove EO. The same research group also reported a similar effect for ginger EO (1.5%) against *E. coli* O157:H7 in Kashar cheese [63]. In the case of ginger EO, the reduction was 4.2 log CFU/g after 30 days of storage in comparison to untreated samples.

The influence of matrix composition is a major challenge for the application of EOs in milk and dairy products. In this sense, the use of coating layers and films seems the most promising approach evaluated in recent studies, which can improve the protection against outbreaks of pathogenic *E. coli*.

### 3.4. Vegetables

The use of EOs against pathogenic *E. coli* is presented in Table 5. The strategies explored by researchers comprise: the use of EOs in washing/rinsing solutions, the incorporation as a food additive, and their incorporation in coating layers and films. Regarding the effect of EOs using the washing/rising solutions, a recent study evaluated the effect of an emulsion containing carvacrol (4000 or 8000 ppm) in broccoli and radish seeds [64]. The authors observed that reductions up to 3.4 log CFU/g were obtained in *E. coli* O157:H7 counts in radish seeds treated with an 8000 ppm carvacrol emulsion after 30 and 60 min of washing. A slight reduction was observed using a 4000 ppm of carvacrol solution treated for 30 min of washing wherein the maximum reduction observed was 2.1 log CFU/g. Moreover, the authors also explored the presence of *E. coli* on sprouted radish seeds and indicated that the pathogen was detected for radish seeds with initial contamination levels higher than 3.4 log CFU/g. However, the *E. coli* on broccoli seeds were not affected by carvacrol EO, regardless of carvacrol concentration and washing times.

A similar outcome was reported for cinnamon bark and leaf EOs (0.25%) to wash basil leaves [65]. In this study, the reduction in *E. coli* O157:H7 was dependent on whether the emulsification of EO was carried out or not and the concentration of EO. Increasing concentrations (from 0.065 to 0.25%) of both EOs were associated with high reduction rates in *E. coli* counts (up to 2.7 log CFU/mL) for both free and emulsified form. The use of oregano EO in combination with ultrasound was associated with a drastic reduction in *E. coli* O157:H7 counts in lettuce leaves below detectable levels using 0.014, 0.018, 0.022, and 0.025% of oregano EO and sonicated washing times of 5 (continuous) and 25 min (2 s on/8 s off for 25 min) [66]. In the case of cinnamon leaf EO (50 ppm) in kale leaves, a slight reduction in *E. coli* counts was obtained (0.6 log CFU/g) [67].

Another relevant aspect related to washing/rinsing of vegetables with EOs is the inhibition of *E. coli* growth during storage. This aspect was evaluated by Park et al. [68] with geranium EO in washing solution to reduce the contamination of *E. coli* O157:H7 in redbor kale, Chinese cabbage, and green mustard. According to the authors, the use of geranium EO in washing solution reduced *E. coli* counts in all vegetables (around 0.7 log CFU/g) for free and emulsified form. During storage of redbor kale, a trend of reduction during storage was observed for emulsified geranium EO while the count for untreated samples remained constant. In the same line of thought, the solo or binary mixture of EOs components were also associated with reduced *E. coli* O157:H7 count in lettuce leaves stored for seven days at 4 °C [69]. In this experiment, the binary mixtures were thymol with eugenol, carvacrol with eugenol, trans-cinnamaldehyde with eugenol, as well as carvacrol. The markable reduction in *E. coli* counts was obtained from thymol with eugenol and carvacrol with eugenol mixtures after seven days (1.5 log CFU/g).

Differently, the study performed by Shahbazi et al. [70] applied *Ziziphora clinopodioides* (0.1 and 0.2%) as food additive against *E. coli* O157:H7 in barley soup. However, the authors indicated reductions around 1 log CFU/mL using both concentrations of EO after seven days of storage in comparison to untreated samples. Furthermore, in soups added of *Ziziphora clinopodioides* EO, the counts of *E. coli* were below detection limits after seven days of storage. Conversely, untreated samples displayed counts of *E. coli* O157:H7 around 2 log CFU/mL after the same period of storage.

The incorporation of EOs in coating layers and films is a relevant strategy to improve the protection against pathogenic *E. coli*. For instance, the use of liposomes loaded with *Artemisia annua* EO in the coating layer and film (2 mg/mL) were used in cherry tomatoes [71]. The authors indicated that both tomatoes coated or wrapped with film displayed counts of *E. coli* O157:H7 3–4 log CFU/g lower than those obtained from untreated samples. It must be taken into account that this study was conducted using temperatures higher than those usually applied in preservation of food, which support the antibacterial effect of *Artemisia annua* EO in both room temperature (25 °C) and on *E. coli* optimum growth temperature (37 °C). Similarly, the use of a carvacrol nanoemulsion in the coating layer reduced the counts of *E. coli* O157:H7 in green beans [72]. According to the authors, the coating with carvacrol nanoemulsion induced a gradual decrease in *E. coli* count during storage down to levels below detection after 11 days of refrigerated storage. Conversely, the count of *E. coli* on untreated green beans displayed a gradual increase until the end of storage.

Collectively, the use of coating layers and films incorporating EOs can be seen as the most consistent and effective strategy against pathogenic *E. coli* in vegetables, but more studies are necessary to support the evidence accumulated so far. Conversely, the use of EOs in washing/rinsing solutions displays a wide range of reductions in pathogenic *E. coli* populations. The use of temperatures above the usually applied to study microbiological stability of refrigerated products (from room temperature to 37 °C) can be seen as additional challenge for the EO. In this sense, some studies provided results that support the potential of EOs on optimal conditions for pathogenic *E. coli* growth. At the same time, exploring this non-usual condition have to be seen as an additional information of scientific importance rather than a potential storage condition in the food chain and retails.

## 4. Conclusions

The use of EO is an interesting strategy that can be seen as an additional barrier against food contaminated with pathogenic *E. coli*. These compounds are largely distributed among plant sources and some relevant compounds can be indicated as main antibacterial compounds (such as thymol, carvacrol, and eugenol). The study of the EO inhibitory effect on Stx production is a topic of major importance to design effective strategies that can directly affect the mechanisms involved in its production. In the case of using sub-MIC concentrations of EOs against pathogenic *E. coli*, the down-regulation of virulence-related genes can take place during lag and exponential phases. However, more evidence is necessary to support this influence/mechanism since this effect was observed in vitro (culture medium) rather than on food systems. Additionally, a contrasting result was obtained between *in vitro* and a food system supporting the hypothesis that matrix can play a major role in the application of EO to inhibit the synthesis of Stx.

Regarding the bacteriostatic and bactericidal effect in food systems, the effect is dependent of EO composition, food matrix composition and processing conditions. Important challenges in the application of EO in free form can be surpassed by the incorporation into an emulsion (when used as a food additive). The most promising strategy to inhibit the growth of pathogenic *E. coli* across food matrixes is by coating or wrapping in a film incorporated of EO.

## Figures and Tables

**Table 1:** Influence of EOs on the expression of genes related to virulence (in vitro) on *E. coli.*

EO (Dosage)	Tested Genes	Effect	Ref.
Oregano and carvacrol, free form (both at 0.05 and 0.08 µL/mL)	*ler*, *stx2B*, and *luxS*	Down-regulation of all tested genes in a concentration-dependent manner for both oregano EO and carvacrol.	[33]
Cinnamon, free form (0.25, 0.50, 0.75 and 1 MIC)	*hfq*, *luxS*, *qseB*, *qseC*, and *stx2*	Down-regulation of *hfq*, *luxS*, *qseB*, *qseC*, and *stx2.*	[29]
*Zataria multiflora*, free and nanoliposomes (25, 50 and 75 of MIC)	*stx2A*	Down-regulation of *stx2A* expression at 50 and 75% MIC.	[30]
Clove and eugenol, free form (both at 0.005%)	*luxS*, *luxR*, *stx1*, *stx2*, *ler*, *espD*, *escJ*, *escR*, and *tir*	Down-regulation of genes related to AEL; no effect on *stx* and quorum-related genes expression.	[31]
*Carum copticum*, free form (0.01–0.04%)	*stx1A* and *stx2A*	Increased the expression of *stx1A* and *stx2A* at 0.03%; down-regulation of *stx1A* and *stx2A* on ground beef.	[32]

EO: essential oil; MIC: minimum inhibitory concentration; AEL: attaching and effacing lesions; *hfq*: associated with PNPase or PAP I (associated with *stx2* phage development); *luxS*, *luxR*, *qseB* and *qseC*: associated with quorum sensing/cell-to-cell communication for pathogenicity induction; and *ler*, *espD*, *escJ*, *escR*, and *tir*: associated with AEL.

**Table 2:** Influence of essential oils on *E. coli* strains in meat and meat products.

Food	EO (Dosage) and Strategy	Processing/Storage Conditions	Effect	Ref.
Fresh turkey sausages	*Mentha suaveolens* EO (2, 5 and 10 mg/g) as additive	48 h at 4 °C	Reduction of 1.2 log CFU/g after 48 h (10 mg/g).	[40]
Beef patties	Clove bud EO (0.3 and 0.5%) as additive	After cooking	Reduction of 0.3 log CFU/g.	[41]
Beef patties	*Ziziphora clinopodioides* (0.1 and 0.2%) as additive	9 days at 4 °C	Reduction of 5.6 log CFU/g after 7 days.	[42]
Fresh pork sausage	Garlic EO (125 and 250 ppm) and allyl isothiocyanate (125 and 250 ppm) mixture as additive	20 days at 6 °C	Reductions of 0.7 and 1.6 log CFU/g after 20 days.	[43]
Salami	Allyl isothiocyanate (3.125 and 6.25 μL/100 g) and *o*-coumaric acid (375 and 750 mg/100 g) as additive	3 days reducing from 26 to 20 °C, 36 h reducing from 20 to 14 °C and 31 days at 14 °C	Reduction of 4.0 log CFU/g during processing.	[44]
Chicken pâté	Oregano EO (0.6 and 12 g/kg) nanoemulsion as additive	8 days at 4 °C	Reduction up to 1.5 log CFU/g after 3 days.	[45]
Fresh chicken breast fillets	Thymol and carvacrol mixture (1:1; 0.4 and 0.8%) in marinating solution	21 days at 4 °C	Reduction up to 0.8 log CFU/g after 21 days.	[46]
Chicken skin	Cinnamon, oregano, and thyme EOs mixture (1%) in marinating solution	24 h at 22 °C	No effect.	[47]
Chicken burger	Anise EO (0.5–2%) in chitosan film	12 days at 4 °C	Reduction of 1.3 log CFU/g after 3 (1–2%) and 6 (0.5%) days.	[48]
Fresh pork meat	Ginger EO (10–80%, EO:protein in film) microencapsulated in a film	9 days at 4 °C	Reductions from 2 to 6 log CFU/g.	[49]
Fresh pork meat	Mexican oregano and basil EOs mixture (4:11; 1.2 mg mixture/cm^2^) microencapsulated in a coating layer	28 days at 4 °C	Reduction of 5.7 log CFU/cm^2^ after 28 days.	[50]

EO: essential oil.

**Table 3:** Influence of essential oils on *E. coli* strains in fish.

Food	EO (Dosage) and Strategy	Processing/Storage Conditions	Effect	Ref.
Rainbow trout (*Oncorhynchus mykiss*) fillet	Oregano and thyme (0.2%) sprayed in the surface	4 days at 4 °C	Reductions up to 1.2 log CFU/g	[51]
Carp (*Cyprinus carpio*) flesh fingers	Rosemary, cinnamon, fennel, and cardamom (10 mL/kg)	14 days at 4 °C	Reduction by up to 1 log CFU/g	[52]
Kachlan (*Trachinotus ovatus*, Linnaeus) fillet	*Pulicaria inuloides* (0.1–0.3 g/100 g) in dipping solution	12 days at 10 °C	Reductions up to 1 log CFU/g	[53]
Rainbow trout (*Oncorhynchus mykiss*) fillet	*Zataria multiflora* and *Bunium persicum* (0.5 and 1%) in a coating layer	12 days at 4 °C	Reductions up to 1 log CFU/g	[54]
Rainbow trout (*Oncorhynchus mykiss*) fillet	*Zataria multiflora* Boiss (0.5 and 1%) in a coating layer	16 days at 4 °C	Reductions around 1.3 log CFU/g	[55]
Silver carp (*Hypophthalmicthys molitrix*) fillet	Clove (1 and 1.5 %) in a coating layer	16 days at 4 °C	Reduction between 3 (1%) and 5 log (1.5%) CFU/g	[56]

EO: essential oil.

**Table 4:** Influence of essential oils on pathogenic *E. coli* in milk and dairy products.

Food	EO (Dosage) and Strategy	Processing/Storage Conditions	Effect	Ref.
Skim, 2% reduced-fat, and full fat milk	Thymol (1 and 4.5 g/L) in free and emulsion as additive	50 h at 21 °C	Reduction up to 6 log CFU/mL, effect was dependent of EO and fat milk contents.	[57]
Kishk	*Teucrium polium* (75 and 150 mg/L) as additive	20 days at 4 °C	Reduction of 0.8 and 1.3 log CFU/g after 20 days.	[58]
Doogh	*Ziziphora clinopodioides* (0.1 and 0.2%) as additive	9 days at 4 °C	Reduction up to 0.3 log CFU/mL after 3 days.	[59]
Iranian white cheese	*Bunium persicum* (1 and 2%) as additive	45 days at 4 °C	Reduction of 0.6 log CFU/g.	[60]
Kariesh cheese	Thyme and clove (0.5 and 1.0%) as additive	14 days at 6 °C	Reductions of 1.8 (1%, clove EO) and 6 (1%, thyme EO). log CFU/g after 14 days	[61]
Kashar cheese	Thyme and clove (1.5%) in coating layer	60 days at 4 °C	Reduction up to 2 log CFU/g after 60 days of storage.	[62]
Kashar cheese	Ginger (1.5%) in coating layer	30 days at 4 °C	Reduction of 4.2 log CFU/g after 30 days of storage.	[63]

EO: essential oil.

**Table 5:** Influence of essential oils on pathogenic *E. coli* in vegetables products.

Food	EO (Dosage) and Strategy	Processing/Storage Conditions	Effect	Ref.
Broccoli and radish seeds	Carvacrol emulsion (4000 or 8000 ppm) in washing solution	30 and 60 min at room temperature	Reduction up to 3.4 log CFU/g (8000 ppm, 60 min); no effect on broccoli seeds.	[64]
Basil leaves	Cinnamon bark and leaf in (0.25%) free and emulsion in washing solution	3 min at room temperature	Reduction of 2.7 log CFU/mL in concentration-dependent manner.	[65]
Lettuce leaves	Oregano (0.010, 0.014, 0.018, 0.022, and 0.025%) in washing solution with ultrasound	Washing on continuous (5 min) or pulsed (2 s on/8 s off for 25 min) mode for ultrasound	Reduction was concentration-dependent was intensified when combined with ultrasound.	[66]
Kale leaves	Cinnamon leaf (50 ppm) in washing solution	3 min at room temperature	Reduction of 0.6 log CFU/g.	[67]
Redbor kale, Chinese cabbage, and green mustard	Geranium (0.1%) in free and emulsion in washing solution	Washing for 3 min at room temperature and storage for 7 days at 4 °C	Reduction around 0.7 log CFU/g for all vegetables after washing; reduction of 1.8 log CFU/g after 7 days on emulsified geranium EO on redbor kale.	[68]
Lettuce leaves	Thymol with eugenol (both at 0.63 mg/mL), carvacrol with eugenol (both at 0.63 mg/mL), trans-cinnamaldehyde with eugenol (0.31 and 1.25 mg/mL), and carvacrol (0.63 mg/mL) in rinsing solution.	7 days at 4 °C	Reduction around 1.5 log CFU/g after 7 days.	[69]
Barley soup	*Ziziphora clinopodioides* (0.1 and 0.2%) as additive	9 days at 4 °C	Reduction of 1 log CFU/mL after 5 days.	[70]
Cherry tomato	*Artemisia annua* in nanoliposomes coating layer and film (2 mg/mL)	10 days at 25 and 37 °C	Reduction between 3 and 4 log CFU/g.	[71]
Green beans	Carvacrol nanoemulsion (0.05%) in a coating layer	13 days at 4 °C	Count below detectable level after 11 days.	[72]

EO: essential oil.
